# “It’s just getting the word out there”: Self-disclosure by people with young-onset dementia

**DOI:** 10.1371/journal.pone.0310983

**Published:** 2024-09-30

**Authors:** Gianna Kohl, Wei Qi Koh, Katrina Scior, Georgina Charlesworth

**Affiliations:** 1 UCL Unit for Stigma Research, Clinical, Educational and Health Psychology, University College London, London, United Kingdom; 2 School of Health and Rehabilitation Sciences, The University of Queensland, Brisbane, Australia; 3 Research and Development, North East London NHS Foundation Trust, London, United Kingdom; The Hong Kong Polytechnic University, HONG KONG

## Abstract

**Background:**

Sharing a dementia diagnosis with others is a prerequisite to accessing important support for social, cognitive, and physical activity. However, due to the stigma associated with dementia, individuals may be hesitant to disclose their diagnosis. Despite the importance of this issue, there is limited research on personal experiences with sharing one’s diagnosis. This study explored how people with young-onset dementia disclose their diagnosis to other people, also known as self-disclosure, and how time affects self-disclosure.

**Methods:**

We conducted an exploratory qualitative study, using semi-structured interviews with nine people with young-onset dementia living in the United Kingdom (UK). A narrative approach to analysis was applied, focusing on understanding the core narratives, themes, tone, and imagery of each participant’s narratives as well as providing a cross-case analysis to identify patterns across narratives.

**Results:**

Participants openly disclosed their diagnosis, accepting it as an illness that did not define their identity. Several were met with stigmatizing reactions, which affected their levels of openness, and a lack of understanding, which caused shrinking social networks for some. Peer support groups, advocacy activities, and strategic concealment were used to support self-disclosure.

**Conclusion:**

This study provides a holistic understanding of people with young-onset dementia’s experiences with self-disclosure and how these evolved. Policies should prioritize the creation of dementia-friendly communities, while recommendations for practice include integrating empowerment interventions and peer support into post-diagnostic support. These efforts will support individuals in their self-disclosure journey, promote social engagement and reduce stigma.

## Introduction

Dementia, an umbrella term for progressive neurodegenerative conditions such as Alzheimer’s disease and vascular dementia, is most prevalent among older adults but also affects younger adults. Young-onset dementia refers to symptom onset and diagnosis before the age of 65 [1]. It presents individuals with unique challenges as their age often means that they face disruptions in crucial roles and responsibilities such as employment, financial commitments, parenting or caring for their own parents [1]. The stigma of dementia can pose additional negative implications for individuals, including feeling and being seen as too young to have dementia [2, 3]. Additionally, a lack of awareness—encompassing symptom-related and age-related misconceptions—among the general public and healthcare professionals about less common forms of dementia such as frontotemporal dementia and posterior cortical atrophy, can negatively impact those affected [4, 5].

Dementia-related stigma, like that of other health conditions, emanates from numerous societal and individual systems that coexist in a dynamic relationship [6]. This stigma is shaped by how both people with dementia and those without it perceive and discuss the condition [7]. In the early stages, the condition and its symptoms can, to an extent, be concealed from other people. Indeed, many individuals in the early stages or with mild symptoms decide not to share their diagnosis due to stigma [8]. While concealing one’s identity or diagnosis may protect individuals from discrimination and stigmatisation, it likely prevents useful accommodations being put in place and can also negatively affect individuals’ psychological and physical health, including leading to avoidance of social situations, increased loneliness, and decreased levels of self-esteem [9].

While the stigma of dementia has been well-documented, research on self-disclosure, that is, the sharing of one’s identity or personal information with others [10], in people with dementia is limited. To our knowledge, only five studies have explored how people with dementia navigate the process of self-disclosing–or choosing not to disclose–their dementia diagnosis to social networks [11–15]. These studies, all of qualitative nature employing diverse methods for data collection (i.e., interviews, group discussions, and observations) and analysis (i.e., grounded theory, qualitative content analysis, deductive analysis, and qualitative analysis not further specified), offer valuable insights. However, none of them have specifically focused on exploring self-disclosure in people with young-onset dementia. Considering the specific needs and experiences of people with young-onset dementia [3], it is important to gain a thorough understanding of individuals’ self-disclosure journey to offer appropriate support to individuals living with the condition. Building on the existing studies’ methodologies and recognizing the need for a comprehensive exploration of the intricate nature of self-disclosure in dementia, the present study adopted a narrative approach to delve deeper into the personal stories of people with young-onset dementia. By allowing participants to share their stories in their own words, a narrative approach offers a unique depth of insight into the complexities of self-disclosure and individual’s’ personal experiences. A narrative approach also offers the opportunity to explore how self-disclosure evolves with time [16], which was partially touched on by Weaks et al. [15]. Filling this important research gap will aid the development of targeted post-diagnostic support and interventions, which can be lacking for people with young-onset dementia [17].

### A narrative approach to dementia

Engaging in storytelling and creating personal narratives enables individuals to construct meaning of the world and communicate their experiences and identities [18]. Narratives offer insights into individuals’ life trajectories over time as they represent more than a mere ‘snapshot’ of someone’s life at a set point in time [19]. Interest in the role of narratives as an approach to understanding the experience of living with illness has grown since the 1980s, and it has been suggested that chronic illness represents a “biographical disruption” [20] to a person’s life, altering one’s sense of self, identity, and relationships.

In recent years, the application of narrative approaches has extended its focus to exploring the unique challenges posed by chronic illness. In dementia research, the majority of existing narrative inquiries seem to have focused on the experiences of family caregivers or couples living with dementia [21, 22]. A few studies have also explored the experiences of people with dementia through their personal narratives [23, 24], with Buggins et al. describing participants’ narratives as “vivid and multi-faceted” [23]. Supporting people with dementia to share their personal stories offers a valuable opportunity for researchers to understand their lived experiences and contribute to a deeper understanding of the dementia journey.

### Study aims

This study aimed to better understand the experience of disclosing or concealing a diagnosis of dementia to and from other people through a narrative inquiry. Specifically, we focused on the following questions:

1. How do people with young-onset dementia experience potential disclosure of their diagnosis on a personal and interpersonal level?
2. How does self-disclosure change over time in people with young-onset dementia?

## Methods

### Study design

This qualitative exploratory study adopted a narrative approach, with semi-structured interviews taking place between February and June 2022. The reporting of this study was guided by the COnsolidated criteria for REporting Qualitative research (COREQ) checklist, see S1 File [25].

### Participants and recruitment

Initially, our aim was to explore self-disclosure in the general dementia population. Due to the overwhelming representation of participants diagnosed before the age of 65, however, we focused specifically on young-onset dementia. Additionally, participants had to be fluent in English and have capacity to provide informed consent. This study used a convenience sample, with recruitment taking place through Join Dementia Research (JDR), a UK-based platform connecting individuals to dementia research, UK-based dementia charities, and social media. Individuals who had participated in another research project and consented to be contacted were emailed to inform them about the study. Recruitment for this study started on 31^st^ January 2022 and ended on 10^th^ June 2022. The researchers of this study were not acquainted with the participants prior to the interviews, and no participants dropped out during the study.

### Procedure

Prospective participants were contacted via email or phone to discuss the consent procedure and interview details, answer any questions they had about the study, and offer them the option to receive the interview questions in advance. Interviews were scheduled to take place approximately one to two weeks after participants received the study details. Participants took part in a single interview, conducted over the phone or online according to their preference due to COVID-19, and each interview was audio-recorded. Field notes were taken after each interview. Participants had the choice to include a family member for support. Demographic information was collected after the interviews had concluded, and participants received a debrief email along with a retail voucher as a token of appreciation.

### Data collection

The interviews were conducted by a female researcher (GK) with experience in qualitative research involving people with dementia. At the time of the study, she was employed as an early-stage researcher at University College London and was pursuing a PhD in psychology. Semi-structured interviews were chosen for this study to balance the depth of qualitative data with the flexibility needed to explore participants’ unique experiences with self-disclosure, allowing them to share their thoughts and feelings freely [26]. We developed a topic guide (S2 File) based on existing research on self-disclosure in stigmatized conditions, consisting of open-ended questions (e.g., “Since you were told you had dementia, what kinds of conversations do you and people who are close to you have about the diagnosis, between yourselves?”). Follow-up questions were asked to gain a greater understanding of the topics discussed. Demographic information collected included age, ethnic group, gender, living situation, type of dementia, and time since diagnosis.

### Epistemological stance

As narrative research is concerned with how individuals construct their own experiences into stories, including their interpretation and meaning making of them, this study took the position that there is no knowable reality or truth that can be ‘uncovered’. Adopting a social constructionist approach, we assume that the ‘reality’ of an individual is dependent on their narratives and not on social and material structures [27]. According to Burr [28], social constructionism is a perspective that assumes that individuals’ knowledge and understanding of the world is shaped by history and culture. Adopting this stance, we aimed to explore and understand how people with dementia have constructed their reality by examining their use of language and narratives to describe their experiences around self-disclosure [29].

### Data analysis

All interviews were transcribed verbatim, and the transcripts were proof-read against the recordings. Participants were given a pseudonym and potentially identifiable information was removed. A narrative analysis was then conducted, based on Crossley’s [30] approach, which was specifically developed to analyze illness-related narratives. The analysis was carried out by two researchers (GK, WQK) in Microsoft Word. After reading transcripts repeatedly, both researchers independently identified the following concepts based on McAdams [31]: (1) narrative tone, which resembles the content of the story as well as how it was told; (2) imagery, which refers to the characteristic set of images that the narratives express; and (3) themes, which resemble the dominant patterns in the narratives. Interpretations of these concepts, along with any discrepancies between the researchers, were discussed with a third researcher (GC). The concepts were then combined into one coherent story for each participant by writing a short narrative summary, drawing on the descriptive phase of Murray’s [32] approach. In doing so, each narrative was ‘restoried’ to place the summaries in chronological order [33]. Lastly, a cross-case analysis was conducted to establish similarities and differences between narratives. Quotes demonstrating these similarities and differences were collected, grouped into three overarching categories, and incorporated into the write-up along with the respective quotes.

### Ethical considerations

Ethical approval was granted by the University College London Research Ethics Committee (ethics ID 1696/001). All participants received oral and written study information prior to taking part and provided informed consent in writing. While dementia can affect a person’s capacity to consent, partly due to fluctuating mental capacity, all participants in this study were able to provide informed consent independently and demonstrated sufficient capacity. To ensure ongoing validity of consent, verbal assent was obtained at the start and during each interview by reviewing key consent details (e.g., participation is voluntary), verifying continued consent, and examining participant engagement throughout the interview.

### Findings

Nine participants with young-onset dementia took part in the study. Their demographic details can be found in Table 1. The mean age was 63.7 years (*SD* = 3.93) and the range of having lived with the diagnosis was one to ten years. Everyone identified as White British, and all but one lived with their spouse, while Rachel lived alone. In the interview with Arthur, his wife clarified points if he struggled finding the right words.

**Table 1:** Characteristics of participants.

Pseudonym	Age	Gender	Type of dementia	Time since diagnosis
Brian	60–64	Male	PCA	5–7 years
Amy	60–64	Female	FTD	>7 years
Rachel	65–69	Female	AD	1–3 years
Heather	55–59	Female	AD	1–3 years
Ruth	65–69	Female	AD & VD	5–7 years
Jane	60–64	Female	AD	5–7 years
Arthur	65–69	Male	FTD	3–5 years
Eileen	65–69	Female	VD	>7 years
Charles	55–59	Male	AD	3–5 years

*Note*. AD = Alzheimer’s disease; FTD = Frontotemporal dementia; PCA = Posterior cortical atrophy; VD = Vascular dementia

### Narrative summaries

Interview duration ranged from 25 to 62 minutes, with a mean of 42 minutes. Each participant presented a unique narrative, with variations in speed and flow. Table 2 provides an overview of the analyses of core narrative, themes, tone, and imagery for each participant. Subsequently, a brief summary of each narrative is given. Words added or revised for context or to ensure anonymity are enclosed in square brackets ([]), whilst an ellipsis (…) indicates omitted text.

**Table 2:** Findings from analysis presenting core narrative, themes, tone, and imagery.

Pseudonym	Core narrative	Themes	Tone	Imagery
Brian	Routinising disclosure	• Mitigating potential misunderstandings • Activism and the notion of equality • Responding to challenging situations	Proud Certain Comfortable	“I see two moons”
Amy	“Caring is sharing”	• Finding a new purpose	Optimistic Hopeful Strong minded	Caregiver
Rachel	“It’s part of me, it’s not all of me”	• Acquiring support through disclosure • Being more than dementia	Strong Defiant Disappointed	“Mummy as usual”
Heather	“You mention that word dementia, and it just changes everything”	• Balancing role loss and keeping a sense of self • Differential attitudes and stigmatization • Navigating life with dementia	Disheartened Uncertain Hopeful	“Swept under the carpet”
Ruth	“I recognize its limitations, but I dismiss it. It’s not part of me”	• Acceptance leads to openness • Positive responses facilitate disclosure • Re-evaluating disclosure	Strong Defiant Capable	“It’s just another ailment”
Jane	“If somebody needs to know, I tell them”	• Supporting myself and others • Natural expansion of disclosure	Calm Independent Strong Encouraging	“Another weight lifted off”
Arthur	“I don’t hide my disability”	• Navigating openness and potentially hurtful reactions • Social withdrawal to maintain dignity • Invisible illness	Disappointed Determined Thoughtful	Sunflower lanyard
Eileen	“I’m pretty much out and proud”	• Keeping silent: Initial shame and embarrassment • Cyclical process of positive reinforcement • Raising awareness through advocacy and charity work	Passionate Assured Defiant	Loud and proud
Charles	“I could sit and talk all day about it”	• New network and purpose • Disclosure for oneself and others Getting the word out there	Cheerful Optimistic Satisfied	“Straight from the horse’s mouth”

*Brian* had routinized self-disclosure. Over the years, he became a vocal activist and was proud to share his experiences. He was not ashamed of his diagnosis and found it merely a “different way of retirement”. Positive reactions from others were an encouragement for him to disclose, although he also experienced negative reactions. While he had no problems sharing his diagnosis, he was more inclined to tell people he saw regularly: “The postman knows because we see him every day. But not the Amazon driver”.

At the time of her diagnosis, *Amy* was working in the healthcare sector but was made redundant. Identifying herself as a caregiver, she was open about her diagnosis to support other people with dementia. In doing so, she was able to carry on her caring function: “I tell people because I can’t change the diagnosis. And if you see someone with a problem, I want to help”. Since dementia is not easily visible, self-disclosure helped her to explain difficulties and receive support.

*Rachel* lives in a small community where the “jungle drums” spread the word about her diagnosis for her. She received support from a health professional who advised her “to be like you are in life anyway, very open”. Sharing her diagnosis with her wider social circle was “difficult” at times, as some people would “say things that are not appropriate or not kind”. She stated that dementia “is [a] part of me, it’s not all of me”, using these words to describe that she “absolutely” did not want to be defined by the diagnosis. She worried about disclosing her diagnosis in the beginning, saying “once I said it, there would be no coming back from it”. However, realizing that she wanted to “explain [her] actions” and that she would gradually need more support led her to share her diagnosis with others.

Initially “feeling very depressed”, *Heather* and her spouse made the decision to disclose the diagnosis openly. Her family reacted with disbelief and friends and acquaintances stopped speaking to her. At appointments, doctors would only address her husband. Overall, she experienced a lot of negative attitudes, with people assuming she was too young to have dementia, and a lack of empathy: “Not one person really has asked me how I was feeling … you can see the shock and horror in people’s faces when you tell them”. To raise awareness and support other people with dementia, she became active on social media. Sharing her diagnosis helped her to accept it: “It’s out in the open and so I can get on and live my life … I don’t have to hide from anybody. I could just be me”.

*Ruth* strongly believed that she is more than her diagnosis. Initially feeling shocked, she quickly discussed the diagnosis with family and friends to move forward and implement support strategies. She started disclosing it more widely to “not let it beat [her]”. She disclosed her diagnosis to everyone she felt was “relevant”, including strangers. Friends were empathetic and supportive. However, people at church reacted dismissively and she was not able to contribute as before. Since then, she disclosed more selectively to avoid having it “affect other people’s opinion of [her] before they get to know [her]”. She described being reluctant to disclose when it was not apparent, as she did not want to be defined by the diagnosis. Ruth became a strong dementia advocate.

*Jane* had been open about her diagnosis since the beginning. She and her family expected the late stages to come “really, really quickly”. Reasons for her openness were to explain symptoms and to receive support. Her disclosure “naturally expanded”, though she said: “I don’t go out shouting it, … but if somebody needs to know, I tell them”. She did not mind if people shared her diagnosis with others. Despite her wishes for support, she found it important that others did not make decisions for her. Reactions from others were positive, but she did not want “sympathy”, as “that’s not why [she’s] telling them”. She started sharing her life with dementia on social media to support others.

Receiving the diagnosis came as a relief to *Arthur* after he was repeatedly misdiagnosed, saying: “I don’t hide my disability”. After telling his immediate family, he disclosed his condition to people in his wider social circle. He could be selective regarding disclosure, with empathy being a quality he looked for. His experiences were mostly positive, though others, including some of his children, reacted with disbelief as the condition was not readily visible. Arthur stopped seeing people who were not understanding: “I want to be somewhere where I can do the things that I like to do in a safe environment with people that know of my condition, are empathic towards it, but not mollycoddling me”. Consequently, he became more socially isolated. His reasons for sharing his diagnosis were to raise awareness about his rare dementia and to stay true to himself as “it’s not fair to myself or to my wife to deny the fact that I have this condition”.

*Eileen* is “out and proud”. She had a difficult time adjusting to the diagnosis, thinking that dementia was for “old people”. Initially, she only discussed the diagnosis with her immediate family, choosing people who “wouldn’t go away and start gossiping about it”. She then trained as a dementia friend and started sharing her story, gradually disclosing her diagnosis to her wider social circle and people outside of it. Disclosing her diagnosis led to people acknowledging her needs and inquiring how she is feeling, which she appreciated. However, she also experienced dismissive reactions: “[They] would say, ‘Oh, but you’re fine … You don’t look like you’ve got dementia’. And now I find these things quite irritating because what does someone with dementia look like?”. Over the years, she became a dementia advocate, stating, “My life is really good … I have a real sense of purpose”.

Initially, *Charles* felt shocked and depressed about his diagnosis, having had to give up his job and driver’s license. His family was supportive but friends “were a different kettle of fish” and many turned away due to the “stigma that surrounds dementia”. Some individuals, including health professionals, did not believe that dementia could occur at a younger age. He was very open about his diagnosis, wearing a disability lanyard in public. He found disclosing his diagnosis “very therapeutic” and hoped that his openness would “make things better for [other] people living with dementia”. He became a vocal dementia activist and set up a local support group.

### Cross-case analysis

While every narrative was unique, there appeared to be some similarities between them, for example, in terms of their experiences with other people’s reactions towards them. An exploration of these through a cross-case analysis generated three cross-case ‘themes’: 1) “It’s just an illness like any other”, 2) changes over time, and 3) impact of disclosure on interpersonal relationships and support.

#### “It’s just an illness like any other”

The first theme represents the acceptance demonstrated in participants’ narratives and their stance against stigma. This was evident in the strong and defiant tone of several of the narratives. While all participants acknowledged their dementia-related impairments, Brian, Jane, Charles, Amy, Heather, and Ruth emphasized that the condition had not altered their personalities and identities. This perspective motivated their decision to openly disclose their diagnosis:

*“I like to do things. Just because one day I’m diagnosed with dementia, I shouldn’t be allowed not to do those things anymore”* (Charles)

*“… I realized that there was no reason for me to keep quiet about it*, *it’s just an illness like any other illness*, *nothing to be ashamed of or anything like that*, *and I just was quite open about it*, *the way you would be open about anything else*.*”* (Ruth)

Rachel, though accepting of her diagnosis, seemed to feel more strongly than the other participants that she did not want her diagnosis to dominate conversations: “I’d rather not be defined by it and I’m very worried about letting myself down as well”.

All participants extensively discussed the stigma attached to dementia, highlighting that the act of disclosing served to raise awareness and confront stigma. Rachel, Heather, Jane, and Eileen drew comparisons between the stigma historically associated with cancer and current dementia stigma. Jane noted, “If somebody had a cancer diagnosis, they wouldn’t actually say the word. And dementia can be a bit like that where I don’t think it should be”. Given that all participants had been diagnosed with young-onset dementia, Brian, Rachel, Heather, and Eileen employed the act of sharing their diagnosis to fulfil a dual role: to increase awareness and challenge the misconception that dementia exclusively affects older individuals, as illustrated by Heather:

*“And there’s lots of people that are under the age of 60 that have dementia, under the age of 50 and 40. And so for me, I wanted to bring more awareness that just because I’ve got dementia doesn’t mean to say I have to sit in a chair. We can still live …”*

Brian and Arthur encountered unique experiences of stigma associated with their rare forms of dementia, as symptoms could differ from the more typical dementia forms like Alzheimer’s disease. This included instances where family members, friends, and sometimes professionals did not believe their diagnosis, with Brian recounting the following situation:

*“… We asked for a care package so the care manager came out and she didn’t believe it … she just said you didn’t need any help so there’s nothing I can do for you, because I was talking and everything else.”*

To raise awareness, Charles, Brian, Heather, Ruth, Eileen, and Jane had taken on active roles as dementia activists and advocates, sharing their personal experiences through social media, TV appearances, and participation in dementia outreach activities. Heather emphasized the importance of “getting the word out there” and “[trying] and make it a more positive experience rather than a negative one”. Similarly, Rachel and Eileen discussed their approaches of addressing derogatory comments about dementia or those affected by it, with Eileen saying, “I do tell people who I don’t think will understand, because I think they need to learn a bit more about it … now I would step in and say, ‘Hang on a minute, that’s not appropriate’”.

Brian, Rachel, Jane, Arthur, and Charles had adopted readily visible strategies in their daily lives to make themselves known as individuals living with a disability. These strategies included the use of a white cane for Brian, dementia identification cards carried by Jane and Rachel, and wearing hidden disability sunflower lanyards by Arthur and Charles. Most of them disclosed their diagnosis in person. Jane and Eileen explained that they had told people who lived further away over the phone, with Eileen additionally having told some people via email. However, Eileen stated that people found it difficult to hear it over the phone “because it is quite a big thing to tell people”.

#### Changes over time

Participants’ attitudes towards disclosure changed over time, ranging from being more secretive in the beginning to becoming very open for some, while others shifted from widely self-disclosing at first to becoming more selective over time. As they gradually came to terms with their diagnosis, Heather and Eileen became more open and confident, as explained by Heather:

*“… We walked out of the clinic feeling very empty … I just went home in a sort of a daze really. So, I couldn’t face telling anybody on that day until I sort of got things worked out in my head … So, it took me a while to sort of pick myself up and think ‘right’, the only way forward now is positive because I don’t want to just sit there and do nothing, because I’ll just deteriorate quicker.”*

While Ruth was initially reluctant to disclose her diagnosis and gradually changed her perspective on her own, Heather was supported by her spouse in deciding to be open about hers. Charles, Eileen, and Rachel received support from health professionals, which helped them shift their mindsets and become more comfortable with and open about their diagnosis, as illustrated by Eileen:

*“When I was first diagnosed, I probably spent about 18 months being really depressed about it and not telling anybody, because I was ashamed and embarrassed. And then a local young-onset worker came … to talk to me about how I was coping … I said to her, ‘Oh, what’s the point? I’m really fed up. What is my purpose now?’ And she advised that I did some training with the Alzheimer’s Society to be a dementia friend. So, I started to do some dementia friends sessions locally, and I realized that actually, my story was different.”*

For Brian, Jane, Eileen, Charles, and Arthur the diagnosis came as a relief, as it provided long-awaited answers for the symptoms they were experiencing. Receiving the diagnosis meant they could explain to others what was going on, as Jane explained, “… to let people know why I was acting the way I was acting”. This felt especially helpful for Brian and Arthur whose dementia symptoms were distinct from the more prevalent dementia forms. For example, Brian’s dementia included visual impairments, which led him to adopt a humorous approach to self-disclosure: “… I usually start off by saying, ‘Oh, I see two moons’, and that gets them going”.

Conversely, over time, Ruth and Arthur had become more selective about whom they would disclose to. Both had experienced a lack of understanding from others, which seemed to have played a role in their decisions, as Ruth explained:

*“I don’t want it to color their opinion of me anymore, because it is a stigma attached to dementia and I found that through the church members, not my close friends, but leaders, church members. And I don’t want to affect other people’s opinion of me before they get to know me. Yeah, get to know me, see what I’m really like and then I might tell them.”*

#### Impact of disclosure on interpersonal relationships and support

All participants spoke about the effect that sharing their diagnosis had had on both existing and newly-formed relationships and the support they received from others. The first people that the participants shared their diagnosis with were family members and close friends. While the majority experienced positive reactions, Brian, Rachel, Arthur, and Heather spoke about how (some) family members responded with disbelief and shock. For example, Brian recounted how his children “didn’t believe it” because “I don’t show signs of memory loss or confusion”. Heather’s narrative was defined by her profound disappointment after having been repeatedly met with negative reactions. Recounting her parents’ reaction, she said:

*“They’re both in their eighties, so they took it pretty badly. And this is where the stigma again comes in with dementia … Well, the question was, ‘How long have you got to live?’… how do you answer that question? Because I don’t know. And I just looked at my mom and I said, ‘How long have you got to live?’ [light laughter], and sort of threw the question back, because nobody knows how long they’ve got to live.”*

Similarly, Rachel, Ruth, Eileen, Heather, and Brian experienced dismissive reactions from others upon disclosing their diagnosis, such as not looking like a person with dementia. Rachel had developed a somewhat defiant demeanor at times. For example, she recalled an instance when acquaintances she met in the street started laughing about her memory problems, which prompted her to fire back at them with a feisty remark.

Charles, Arthur, Heather, and Brian also reflected on reactions of individuals who had been close to them prior to the diagnosis but had, as Charles put it, “just stopped coming around”. In contrast to the other participants, these four lost some or all of their friends after they shared their diagnosis. Brian made the decision to resign from a social club, because its members were dismissive and lacked understanding. As a result, he said, “I have withdrawned [sic] myself and in some respects have become more isolated”. Charles speculated that “the whole diagnosis process just scared them off”. Ruth said that she did not get asked to actively participate in church activities anymore, saying, “I feel quite excluded and as by association, my husband feels the same”. She said regretting having disclosed her condition in church. Despite these challenges, their narratives were optimistic and confident, as Brian remarked, “it’s their problem not mine”.

Brian, Amy, Charles, Jane, Heather, Arthur, and Ruth highlighted the importance of peer support groups. These groups not only offered valuable support but also provided a platform for forging connections with like-minded people. Brian captured this feeling of close-knit support, saying, “We all understand each other and protect each other”. Brian, Heather, Ruth, Charles and Amy cited wanting to support other people with dementia as a motivating factor for sharing their diagnosis, as highlighted by Amy: “If you meet somebody who’s newly diagnosed with it, or something like it, then you’re helping them to learn to live with it”.

Rachel, Ruth, and Jane shared that disclosing within their social networks facilitated opportunities for others to offer support. Jane underscored this by saying, “… if you don’t tell people you’re not giving them permission to help … then they don’t know is it okay to ask [Jane] if she’s okay”. However, she stressed her preference for choice-based support: “… the thing I’ve always, always told people is ‘Don’t presume you know what I need, ask me’”. Rachel and Jane talked about disclosing their diagnosis to staff working in local shops or on public transport if they needed support, with Rachel noting, “… they’re always extremely helpful and kind”.

Among all participants, only Rachel recalled having actively concealed her diagnosis. She had developed a close friendship with someone and believed it had the potential to evolve into a romantic relationship but decided to not pursue it any further once she was diagnosed “because that wouldn’t be fair to somebody you were in a relationship with’. In addition, Rachel, Heather, and Brian discussed instances in which they made conscious efforts to conceal their symptoms when in the presence of others, particularly family members. However, these efforts would leave them feeling tired, as illustrated by Rachel’s quote:

*“I can see with [child], he gets quite distressed so I find I feel exhausted when I come away from there because I try so, so hard not to do something silly or let the side down … You sort of play acting in a way. Covering it, which of course compounds the problem, because then they think you’re fine. You become your own victim of your own successful acting skills.”*

## Discussion

To our knowledge, this is the first study to explore experiences of self-disclosure in people with young-onset dementia, including changes over time in their openness. Additionally, this study contributes to the field by applying a narrative approach to analysis, which has rarely been used in research involving people with dementia. This approach allowed for the detailed presentation of individual stories and facilitated a comparison of experiences. The analysis revealed similarities and differences in the narratives regarding experiencing dementia as ‘an illness like any other’, the effect of time on changes in disclosure, and the impact of disclosure on interpersonal relationships.

### Principal findings

Contrary to a previous qualitative study [12], all participants in this study had disclosed their diagnosis, offering a unique insight into self-disclosure in people with young-onset dementia. The findings illustrate participants’ experiences of self-disclosure as a personal and multifaceted journey. Their narratives highlighted the importance and their ability of preserving their identity amid the challenges posed by dementia. This study contributes to the ongoing debates about the persistence of the self in people with dementia [34] by demonstrating that participants exhibited a strong determination to stay true to themselves. Similar to Birt et al.’s [35] ethnographic study, participants accepted their diagnosis by viewing dementia as one part of their lives rather than the dominant one. Over time, this acceptance allowed several participants to disclose their diagnosis more widely within their social networks and beyond. Diagnosis acceptance has been suggested to play a pivotal role in people with dementia preserving their identities [36]. While a qualitative study conducted with people with young-onset dementia proposed a consistent loss of the self [37], this study uniquely found that participants felt confident in themselves, despite adjusting to changes caused by their condition. This underscores the importance of recognizing individuals’ identities beyond their condition or diagnosis.

Participants shared their views on the stigma associated with dementia, with several actively working to confront it. This proactive stance is similar to that described in a recent qualitative study involving people with young-onset dementia [38], where participants aimed to challenge the misconceptions that dementia is related to ‘old age’ and ‘an invisible illness’. Unlike Goffman’s concept of ‘discreditable’ stigma [39], which suggests that individuals might conceal invisible stigmas to avoid social devaluation, our findings illustrate a form of stigma management or resistance [40], where participants sought to reframe the understanding of their diagnosis by raising awareness and dispelling myths. This aligns with the broader literature on health-related stigma and self-disclosure, which recognizes the agency of individuals in addressing stigma through disclosure [14, 41–43]. While some literature suggests that concealing a stigmatized condition might protect individuals from harm [44], our study demonstrates that disclosure can be a strategy to challenge stereotypes. For example, participants openly disclosed their diagnosis to unfamiliar people or used strategies like wearing a disability lanyard, a method previously described as helpful for people with dementia [38]. This approach to stigma aligns with discussions in mental health contexts, where visibility and openness are seen as empowering rather than vulnerable [45].

Another approach for participants to combat stigma included taking on roles as activists. Active advocacy aligns with the broader discourse on the role of people with dementia as agents of change in shaping public perceptions, policies, and interventions [46]. In addition, the participants in this study seemed to have ‘blossomed’ in their new roles as dementia advocates. A recent systematic review found that dementia advocacy can provide people with dementia with a strong sense of purpose, enabling them to make contributions beyond themselves [47]. This seems particularly relevant for people with young-onset dementia who are often confronted with additional aspects of loss such as loss of employment [2]. As such, this study demonstrates how advocacy provided participants with a renewed sense of purpose and identity.

The participants’ narratives also revealed complex dynamics within interpersonal relationships following diagnosis disclosure. While many received understanding and support, some encountered disbelief and shock, aligning with previous research on the varied reactions to a dementia diagnosis [48]. Loss of social connections and lack of empathy following disclosure were experienced by some participants in this study. Our findings build on existing research, emphasizing that negative reactions from others to disclosure can lead to increased sensitivity in future self-disclosure in line with the Disclosure Process Model by Chaudoir and Fisher [10], which suggests that the recipient’s reaction influences future self-disclosure. Indeed, some participants in this study had become more selective in their disclosure due to negative reactions from others, similar to findings that were described by Weaks et al. [15].

Several participants found understanding and support through involvement in peer support groups, which can enrich the post-diagnostic experiences of people with young-onset dementia [49]. These groups facilitated support and new connections, a beneficial feature described as a sense of ‘collective strength’ in a previous interview study [50]. Participants who were initially open about their diagnosis but encountered negative reactions found a supportive community in these groups. Our findings highlight that peer support groups were essential in managing stigma and maintaining social connections, thereby enhancing participants’ social and emotional well-being. They also underscore the need for greater societal understanding and address the strain stigma and fear can place on existing relationships [51], while demonstrating the positive impact of peer support networks in fostering a sense of belonging in people with dementia.

### Strengths and limitations

This is the first study to explore self-disclosure in people with young-onset dementia using a narrative approach, offering a rich and unique insight and detailed image of participants’ personal experiences. While this study adds to the body of dementia research that includes the voices of people with dementia, it has limitations.

Firstly, all participants shared a similar socio-economic and ethnic background. While narrative research does not aim to produce generalizable findings, future research would benefit from a more diverse sample encompassing various backgrounds and cultures, as individuals from different ethnic backgrounds may experience dementia-related stigma differently [52].

Secondly, we acknowledge that the experiences of our participants may differ from those of individuals with late-onset dementia. While not initially planned, all but one individual interested in participating had been diagnosed with young-onset dementia. One possible explanation could be the requirement of remote data collection due to COVID-19 regulations at the time, give that age can influence the acceptance of technologies used for this study’s data collection method [53]. However, it may also suggest that people with young-onset dementia face unique challenges related to self-disclosure. This is particularly relevant, considering that most participants in this study referred to the stigma associated with being diagnosed at a younger age. Future studies would benefit from exploring self-disclosure in people with late-onset dementia.

Thirdly, despite efforts to diversify levels of openness, all participants were comfortable disclosing their diagnosis as needed. A potential bias in self-disclosure research due to studies attracting participants who are more open about their diagnosis has been described previously [54]. Future research could consider purposive sampling to explore experiences and narratives of individuals who are hesitant to disclose.

### Implications

The findings from this study provide implications for health and social care professionals, policymakers, and society at large. As revealed in the narratives, disclosure is complex and not always straightforward. Negative reactions, such as disbelief from family, friends, the wider community, and even health professionals, are common and can, in some cases, contribute to social isolation. Individuals not affected by dementia as well as health and social care professionals should be supported in enhancing their awareness and understanding of the various forms and symptoms of dementia, including those that can affect younger people. Furthermore, it is important to recognize that adjusting to the diagnosis can be a staged process that involves many emotions [55]. Health and social care professionals should be aware of this and provide individuals and their families with appropriate post-diagnostic resources and services to support them through the disclosure process and their dementia journey. This support might include interventions that empower people with dementia in their disclosure process, such as the ‘Who to tell, how and when’ intervention [56], as well as signposting to peer support networks like the UK-based DEEP network [57]. Policymakers are urged to prioritize the development of dementia-friendly communities to support and enable people with dementia to remain engaged and active citizens, similar to initiatives undertaken in the UK [58]. When designing services for individuals who are adjusting to a new diagnosis and considering how to share it with their social networks, it is not only important to acknowledge the expertise of people with dementia, but also to actively incorporate it. Conducting research *with* instead of *about* people with dementia aligns with an approach that is respectful of their continuing citizenship [36].

Additionally, the narratives highlighted misconceptions about dementia in general and young-onset dementia in particular. The experiences of participants actively engaged in advocacy and awareness-raising highlight the importance of reducing stigma and empowering people with dementia [59]. This underscores the ongoing need for initiatives that aim to increase dementia awareness and reduce stigma [60]. Research into the effectiveness of these interventions and programs is warranted to gather evidence-based insights into how to best foster awareness and promote acceptance of dementia within society.

## Conclusion

People with young-onset dementia undergo a personal and evolving journey in managing disclosure of their diagnosis. At its core is the wish to stay true to oneself and preserve one’s identity while adapting to changes associated with the condition. Over time, most participants became more open or selective about sharing their diagnosis, influenced by reactions from others and a desire to confront stigma. Additionally, participants leaned on significant others and professionals to help them become more comfortable with self-disclosure. The findings provide a holistic understanding of how people with young-onset dementia navigate self-disclosure in their lives, as well as highlighting the need for formal and informal post-diagnostic support in the self-disclosure process.

## Supporting information

S1 File COREQ checklist. (DOCX)

S2 File Interview topic guide. (DOCX)

## References

[1] Rossor MN, Fox NC, Mummery CJ, Schott JM, Warren JD. The diagnosis of young-onset dementia. Lancet Neurol. 2010 Aug;9(8):793–806. doi: 10.1016/S1474-4422(10)70159-9 20650401 PMC2947856

[2] Greenwood N, Smith R. The experiences of people with young-onset dementia: A meta-ethnographic review of the qualitative literature. Maturitas. 2016;92:102–9. doi: 10.1016/j.maturitas.2016.07.019 27621246

[3] Rabanal LI, Chatwin J, Walker A, O’Sullivan M, Williamson T. Understanding the needs and experiences of people with young onset dementia: A qualitative study. BMJ Open. 2018;8(10):e021166.-e021166. doi: 10.1136/bmjopen-2017-021166 30344167 PMC6196838

[4] McIntyre A, Harding E, Yong KXX, Sullivan MP, Gilhooly M, Gilhooly K, et al. Health and social care practitioners’ understanding of the problems of people with dementia-related visual processing impairment. Health Soc Care Community. 2019;27(4):982–90. doi: 10.1111/hsc.12715 30737853 PMC6618310

[5] Griffin J, Oyebode JR, Allen J. Living with a diagnosis of behavioural-variant frontotemporal dementia: The person’s experience. Dementia. 2016 Nov;15(6):1622–42. doi: 10.1177/1471301214568164 25645139

[6] Pescosolido BA, Martin JackK. The Stigma Complex. Annu Rev Sociol. 41:87–116. 10.1146/annurev-soc-071312-145702 PMC4737963 26855471

[7] Meisenbach RJ. Stigma Management Communication: A Theory and Agenda for Applied Research on How Individuals Manage Moments of Stigmatized Identity. J Appl Commun Res. 38(3):268–92.

[8] Lopez RP, Rose KM, Kenney L, Sanborn V, Davis JD. Managing shame: A grounded theory of how stigma manifests in families living with dementia. J Am Psychiatr Nurses Assoc. 2020 Mar;26(2):181–8. doi: 10.1177/1078390319832965 30866693

[9] Quinn DM, Weisz BM, Lawner EK. Impact of active concealment of stigmatized identities on physical and psychological quality of life. Soc Sci Med. 2017;192:14–7. doi: 10.1016/j.socscimed.2017.09.024 28941787

[10] Chaudoir SR, Fisher JD. The Disclosure Processes Model: Understanding disclosure decision making and postdisclosure outcomes among people living with a concealable stigmatized identity. Psychol Bull. 2010;136(2):236–56. doi: 10.1037/a0018193 20192562 PMC2922991

[11] Glavind IML. Questions of (non)Disclosure among People Living with Alzheimer’s Disease in Denmark. Anthropol Aging. 2023;44(3):1–15.

[12] Hellström I, Torres S. A wish to know but not always tell–couples living with dementia talk about disclosure preferences. Aging Ment Health. 2013 Mar;17(2):157–67. doi: 10.1080/13607863.2012.742491 23171298

[13] Kohl G, Koh WQ, Scior K, Charlesworth G. Self-Disclosure and Social Media Use among Younger and Older People with Dementia: An Internet-Mediated Mixed-Methods Study. Int J Hum-Comput Interact. 2023.

[14] O’Connor D, Mann J, Wiersma E. Stigma, discrimination and agency: Diagnostic disclosure as an everyday practice shaping social citizenship. J Aging Stud. 2018;44:45–51. doi: 10.1016/j.jaging.2018.01.010 29502789

[15] Weaks D, Wilkinson H, McLeod J. Daring to tell: The importance of telling others about a diagnosis of dementia. Ageing Soc. 2015;35(4):765–84.

[16] Sparkes AC. Narrative analysis: exploring the whats and hows of personal stories. In: Holloway I, editor. Qualitative Research in Health Care. Open University Press; 2005. p. 191–209.

[17] Cations M, Withall A, Horsfall R, Denham N, White F, Trollor J, et al. Why aren’t people with young onset dementia and their supporters using formal services? Results from the INSPIRED study. Fuh JL, editor. PLOS ONE. 2017 Jul 19;12(7):e0180935. 10.1371/journal.pone.0180935 PMC5517136 28723931

[18] Riessman CK. Narrative Methods for the Human Sciences. Sage; 2008.

[19] Carless D, Douglas K. Narrative research. J Posit Psychol. 2017 May;12(3):307–8.

[20] Bury M. Chronic illness as biographical disruption. Sociol Health Illn. 1982;4(2):167–82. doi: 10.1111/1467-9566.ep11339939 10260456

[21] Brooks A, Farquharson L, Burnell K, Charlesworth G. A narrative enquiry of experienced family carers of people with dementia volunteering in a carer supporter programme. J Community Appl Soc Psychol. 2014 Nov;24(6):491–502.

[22] Tretteteig S, Vatne S, Rokstad AMM. Meaning in family caregiving for people with dementia: A narrative study about relationships, values, and motivation, and how day care influences these factors. J Multidiscip Healthc. 2017 Dec;10:445–55. doi: 10.2147/JMDH.S151507 29263675 PMC5726371

[23] Buggins SL, Clarke C, Wolverson E. Resilience in older people living with dementia–A narrative analysis. Dementia. 2021 May;20(4):1234–49. doi: 10.1177/1471301220927614 32460547

[24] Lea E, Synnes O. “I still want to be part of the world…where I belong.” A case study of the experiences of a man with Alzheimer’s of dementia-friendly guided tours at an art museum. Int J Ageing Later Life. 2021 Sep;15(1):163–91.

[25] Tong A, Sainsbury P, Craig J. Consolidated criteria for reporting qualitative research (COREQ): a 32-item checklist for interviews and focus groups. Int J Qual Health Care. 19(6):349–57. doi: 10.1093/intqhc/mzm042 17872937

[26] Adams WC. Conducting Semi-Structured Interviews. In: Newcomer KE, Hatry HP, Wholey JS, editors. Handbook of Practical Program Evaluation. Wiley; p. 492–505.

[27] Silver J. Narrative psychology. In: Willig C, editor. Introducing Qualitative Research in Psychology. 3rd ed. Open University Press; 2013. p. 143–55.

[28] Burr V. Social Constructivism. 3rd ed. Routledge; 2015.

[29] Willig C. What Can Qualitative Psychology Contribute to Psychological Knowledge? Psychol Methods. 2019;24(6):796–804. doi: 10.1037/met0000218 31008623

[30] Crossley M. Analysing Qualitative Data in Psychology. In: Lyons E, Coyle A, editors. Analysing Qualitative Data in Psychology. London: SAGE Publications; 2007. p. 131–44.

[31] McAdams DP. The Stories We Live By: Personal Myths and the Making of the Self. New York: William Morrow & Co; 1993.

[32] Murray M. Narrative Psychology. In: Smith JA, editor. Qualitative Psychology: A Practical Guide to Research Methods. 3rd ed. SAGE Publications; 2015. p. 85–107.

[33] Cresswell JW. Five Qualitative Approaches to Inquiry. In: Qualitative Inquiry and Research Design. 2nd ed. SAGE Publications; 2007. p. 53–84.

[34] Caddell LS, Clare L. The impact of dementia on self and identity: A systematic review. Clin Psychol Rev. 2010;30(1):113–26. doi: 10.1016/j.cpr.2009.10.003 19896760

[35] Birt L, Charlesworth G, Moniz-Cook E, Leung P, Higgs P, Orrell M, et al. ‘The Dynamic Nature of Being a Person’: An Ethnographic Study of People Living With Dementia in Their Communities. The Gerontologist. 2023 Sep;63(8):1320–9. doi: 10.1093/geront/gnad022 36879407 PMC10474587

[36] Birt L, Poland F, Csipke E, Charlesworth G. Shifting dementia discourses from deficit to active citizenship. Sociol Health Illn. 2017;39(2):199–211. doi: 10.1111/1467-9566.12530 28177147

[37] Busted LM, Nielsen DS, Birkelund R. “Sometimes it feels like thinking in syrup”–the experience of losing sense of self in those with young onset dementia. Int J Qual Stud Health Well-Being. 2020 Jan;15(1):1734277–1734277. doi: 10.1080/17482631.2020.1734277 32111147 PMC7067159

[38] Tingley D, Ashworth R, Torres Sanchez D, Mac Mahon GH, Kusel Y, Rae BM, et al. Is the Invisibility of Dementia a Super-Power or a Curse? A Reflection on the SUNshiners’ Questionnaire into the Public Understanding of Dementia as an Invisible Disability: A User-Led Research Project. Int J Environ Res Public Health [Internet]. 2024;21(4). Available from: https://www.mdpi.com/1660-4601/21/4/466 doi: 10.3390/ijerph21040466 38673377 PMC11050154

[39] Goffman E. Stigma: Notes on the Management of Spoiled Identity. Simon and Schuster; 1963.

[40] Thoits PA. “I’m Not Mentally Ill”: Identity Deflection as a Form of Stigma Resistance. J Health Soc Behav. 2016 Jun;57(2):135–51. doi: 10.1177/0022146516641164 27284073

[41] Bril-Barniv S, Moran GS, Naaman A, Roe D, Karnieli-Miller O. A Qualitative Study Examining Experiences and Dilemmas in Concealment and Disclosure of People Living With Serious Mental Illness. Qual Health Res. 2017 Mar;27(4):573–83. doi: 10.1177/1049732316673581 28682733

[42] Yokoyama K, Morimoto T, Ichihara-Takeda S, Yoshino J, Matsuyama K, Ikeda N. Relationship between self-disclosure to first acquaintances and subjective well-being in people with schizophrenia spectrum disorders living in the community. Denis F, editor. PLOS ONE. 2019 Oct 16;14(10):e0223819. 10.1371/journal.pone.0223819 PMC6795466 31618271

[43] Rohde JA, Wang Y, Cutino CM, Dickson BK, Bernal MC, Bronda S, et al. Impact of Disease Disclosure on Stigma: An Experimental Investigation of College Students’ Reactions to Inflammatory Bowel Disease. J Health Commun. 2018 Jan 2;23(1):91–7. doi: 10.1080/10810730.2017.1392653 29283816

[44] Camacho G, Reinka MA, Quinn DM. Disclosure and concealment of stigmatized identities. Curr Opin Psychol. 2020;31:28–32. doi: 10.1016/j.copsyc.2019.07.031 31430614

[45] Corrigan PW, Kosyluk KA, Rüsch N. Reducing Self-Stigma by Coming Out Proud. Am J Public Health. 2013 May;103(5):794–800. doi: 10.2105/AJPH.2012.301037 23488488 PMC3698841

[46] Hagan RJ, Campbell S. Doing their damnedest to seek change: How group identity helps people with dementia confront public stigma and maintain purpose. Dementia. 2021 Oct;20(7):2362–79. doi: 10.1177/1471301221997307 33599520 PMC8564231

[47] Weetch J, O’Dwyer S, Clare L. The involvement of people with dementia in advocacy: a systematic narrative review. Aging Ment Health. 2021;25(9):1595–604. doi: 10.1080/13607863.2020.1783512 32578451

[48] Pipon-Young FE, Lee KM, Jones F, Guss R. I’m not all gone, I can still speak: The experiences of younger people with dementia. An action research study. Dementia. 2012;11(5):597–616.

[49] Gerritzen EV, Kohl G, Orrell M, McDermott O. Peer support through video meetings: Experiences of people with young onset dementia. Dementia. 2023;22(1):218–34. doi: 10.1177/14713012221140468 36400741 PMC9772896

[50] Clare L, Rowlands JM, Quin R. Collective strength: The impact of developing a shared social identity in early-stage dementia. Dementia. 2008 Feb;7(1):9–30.

[51] Rewerska-Juśko M, Rejdak K. Social Stigma of People with Dementia. J Alzheimers Dis. 2020;78(4):1339–43. doi: 10.3233/JAD-201004 33185610

[52] Herrmann LK, Welter E, Leverenz J, Lerner AJ, Udelson N, Kanetsky C, et al. A systematic review of dementia-related stigma research: Can we move the stigma dial? Am J Geriatr Psychiatry. 2018;26(3):316–31. doi: 10.1016/j.jagp.2017.09.006 29426607

[53] Lythreatis S, Singh SK, El-Kassar AN. The digital divide: A review and future research agenda. Technol Forecast Soc Change. 2022 Feb;175:121359–121359.

[54] Bhatt J, Kohl G, Scior K, Charlesworth G, Muller M, Dröes RM. Comparing the stigma experiences and comfort with disclosure in Dutch and English populations of people living with dementia. Dementia. 2023;22(7):1567–85. doi: 10.1177/14713012231188503 37480343 PMC10521157

[55] Robinson L, Gemski A, Abley C, Bond J, Keady J, Campbell S, et al. The transition to dementia—individual and family experiences of receiving a diagnosis: a review. Int Psychogeriatr. 2011;23(7):1026–43. doi: 10.1017/S1041610210002437 21281553

[56] Bhatt J, Ruffell TO, Scior K, Charlesworth G. “Who to Tell, How and When?”: Development and preliminary feasibility of an empowerment intervention for people living with dementia who are fearful of disclosing their diagnosis. Clin Interv Aging. 2020;15:1393–407. doi: 10.2147/CIA.S257317 32884249 PMC7439500

[57] DEEP. About DEEP [Internet]. [cited 2024 Aug 13]. Available from: https://www.dementiavoices.org.uk/about-deep/

[58] Buckner S, Darlington N, Woodward M, Buswell M, Mathie E, Arthur A, et al. Dementia Friendly Communities in England: A scoping study. Int J Geriatr Psychiatry. 2019;34(8):1235–43. doi: 10.1002/gps.5123 30993756 PMC6766876

[59] Alzheimer’s Disease International. World Alzheimer Report 2019: Attitudes to dementia [Internet]. London; 2019. Available from: https://www.alz.co.uk/research/world-report-2019

[60] Bacsu JD, Johnson S, O’Connell ME, Viger M, Muhajarine N, Hackett P, et al. Stigma Reduction Interventions of Dementia: A Scoping Review. Can J Aging Rev Can Vieil. 2022 Jun;41(2):203–13. doi: 10.1017/S0714980821000192 34253273

