# Supplementary figures and images for: “It’s just getting the word out there”: Self-disclosure by people with young-onset dementia
Source: PLoS One. 2024 Sep 30;19(9):e0310983. doi: 10.1371/journal.pone.0310983 (PMC11441687; doi:10.1371/journal.pone.0310983)

**S1 File. COREQ checklist.**


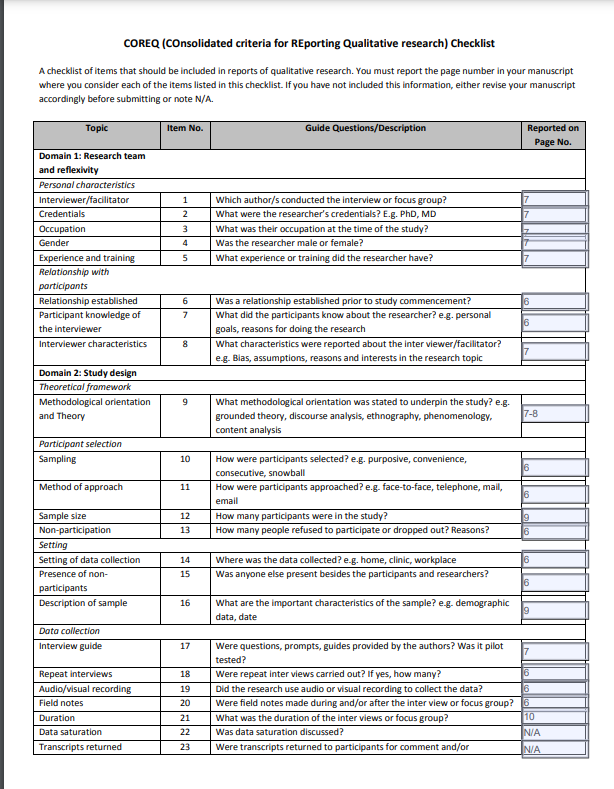


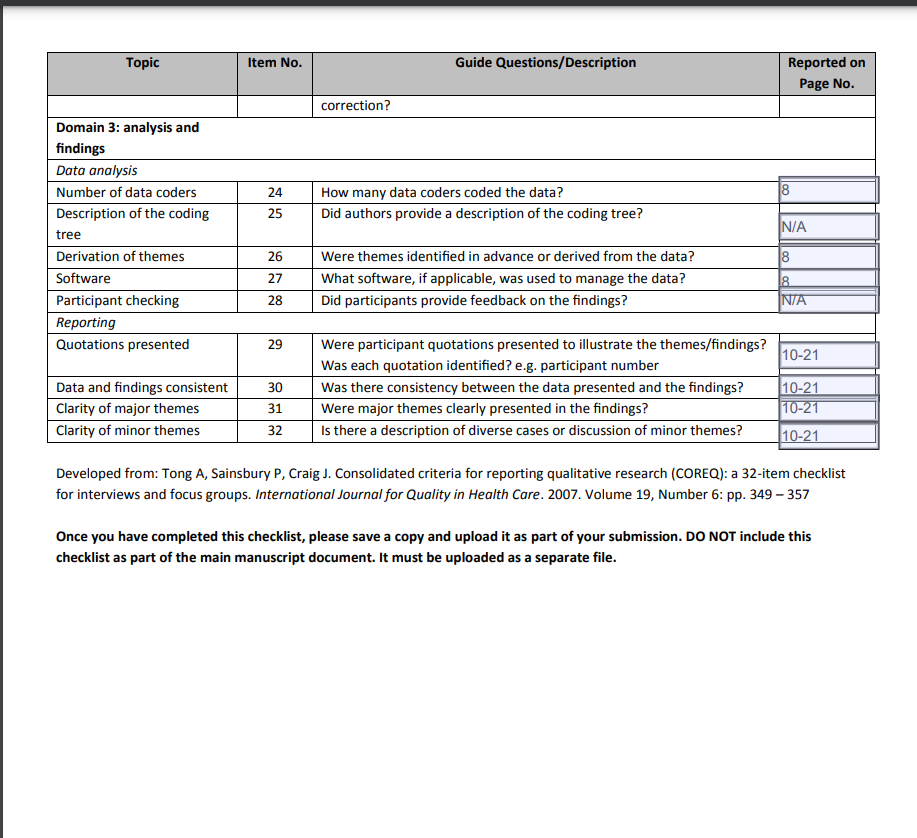

Supplement: S1 File — (DOCX) [file pone.0310983.s001.docx]
