# Supplementary material for: “It’s just getting the word out there”: Self-disclosure by people with young-onset dementia
Source: PLoS One. 2024 Sep 30;19(9):e0310983. doi: 10.1371/journal.pone.0310983 (PMC11441687; doi:10.1371/journal.pone.0310983)
Supplement: S2 File — (DOCX) [file pone.0310983.s002.docx]

**S2 File. Interview topic guide.**

**“It's just getting the word out there”: Self-disclosure by people with young-onset dementia**

1. Since you were told you had dementia, what kinds of conversations do you and people who are close to you, for example your partner/spouse or friends, have about the diagnosis?
2. Have you told other people about your diagnosis?
3. Have you ever decided not to tell others about your dementia?

- When in the company of others, is there anything you do to ‘cover up’ or hide your diagnosis of dementia?

1. What has influenced your decision to tell others or not tell others about your dementia? Why did you decide to tell others about your dementia?
2. What was your experience with telling other people about your dementia?

- What concerns did you have?
- What reactions have you had from others?

1. How has telling others affected your own wellbeing?

- How has this affected your day-to-day routine or levels of support?

1. What kind of support have you had around making the decision to tell others or not tell others?
2. How has your decision to tell others changed over time?
